# Supplementary material for: Structural basis for recruitment of TASL by SLC15A4 in human endolysosomal TLR signaling
Source: Nat Commun. 2023 Oct 20;14:6627. doi: 10.1038/s41467-023-42210-9 (PMC10589346; doi:10.1038/s41467-023-42210-9)
Supplement: Supplementary file 1 — Supplementary Information [file 41467_2023_42210_MOESM1_ESM.pdf]

Supplementary Figures

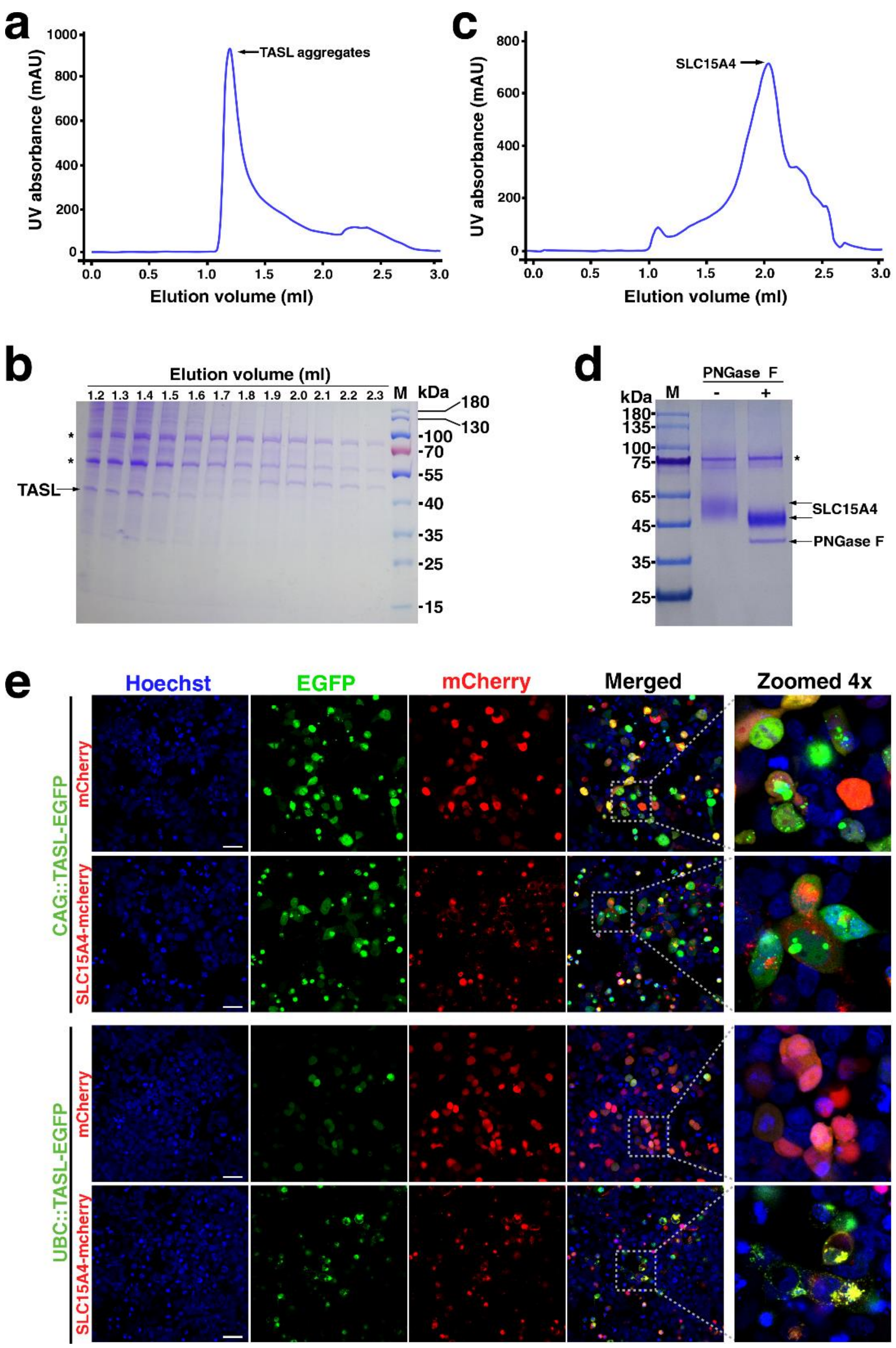

Supplementary Fig. 1 Protein purification of human SLC15A4 and TASL

a, A representative trace of size-exclusion chromatography of human full-length TASL by Superose 6 5/150 GL column. UV, ultraviolet. Data are representative of two independent experiments.

b, The fractions of 1.2-2.3 mL (elution volume) in (**a**) were subjected to sodium dodecyl sulfate polyacrylamide gel (SDS-PAGE). M, marker.

c, A representative trace of size-exclusion chromatography of human full-length SLC15A4 by Superose 6 5/150 GL column. UV, ultraviolet. Data are representative of three independent experiments.

d, The peak frections of protein samples in (**c**) were subjected to SDS-PAGE.

e, Confocal microscopy images of TASL-EGFP (with strong or weak protomer) co-transfected with and mCherry or SLC15A4-mCherry in HEK293T cells. Scale bars, 40 μm. Source data for relevant information are provided as a Source Data file.

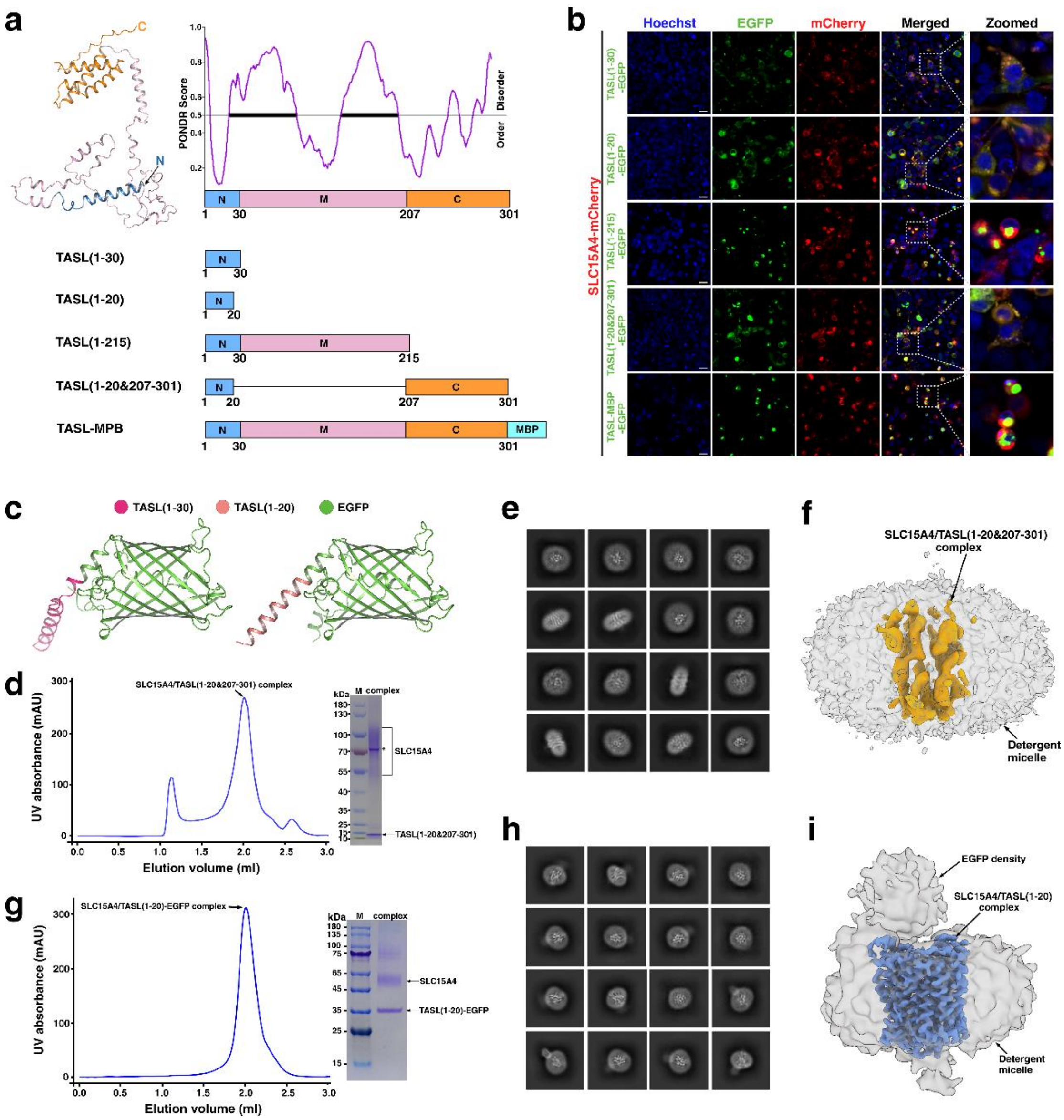

Supplementary Fig. 2 Protein purification and structure determination of human SLC15A4-TASL complex

a, Upper left: The structure prediction of human full-length TASL based on RoseTTAFold[1]. Upper right: Predictions of the disorder regions of TASL based on Predictor of Natural Disorder Regions (PONDR)[2]. The ordered N- terminal and C-terminal regions, as well as the disordered middle region of human TASL are abbreviated N, C and M, and colored blue, orange and pink, respectively. Bottom: The five TASL truncations designed for protein expression.

b, Confocal microscopy images of HEK293T cells co-transfected with SLC15A4-mCherry and the indicated TASL-truncations-EGFP plasmids. Localization of

SLC15A4 and TASL truncations are shown. Data are representative of two-three biological independent experiment. Scale bars: 40 μm.

c, The structure predictions of TASL(1-30)-EGFP and TASL(1-20)-EGFP fusion proteins. The structure predictions are based on RoseTTAFold[1].

d, Left: A representative trace of size-exclusion chromatography of human SLC15A4/TASL(1-20&207-301) complex by Superose 6 5/150 GL column. UV, ultraviolet. Data are representative of three independent experiments. Right: The peak frections of protein samples were subjected to SDS-PAGE. M, marker.

e, 2D class averages of the SLC15A4/TASL(1-20&207-301) complex.

f, 3D density map of the SLC15A4/TASL(1-20&207-301) complex. The density of detergent micelle and protein are colored gray and orange, respectively.

g, Left: A representative trace of size-exclusion chromatography of human SLC15A4/TASL(1-20)-EGFP complex by Superose 6 5/150 GL column. Data are representative of four independent experiments. Right: The peak fractions of the protein samples were subjected to SDS-PAGE.

h, 2D class averages of the SLC15A4/TASL(1-20)-EGFP complex.

i, 3D density map of the SLC15A4/TASL(1-20)-EGFP complex. The density of detergent micelle and protein are colored gray and blue, respectively. Source data for relevant information are provided as a Source Data file.

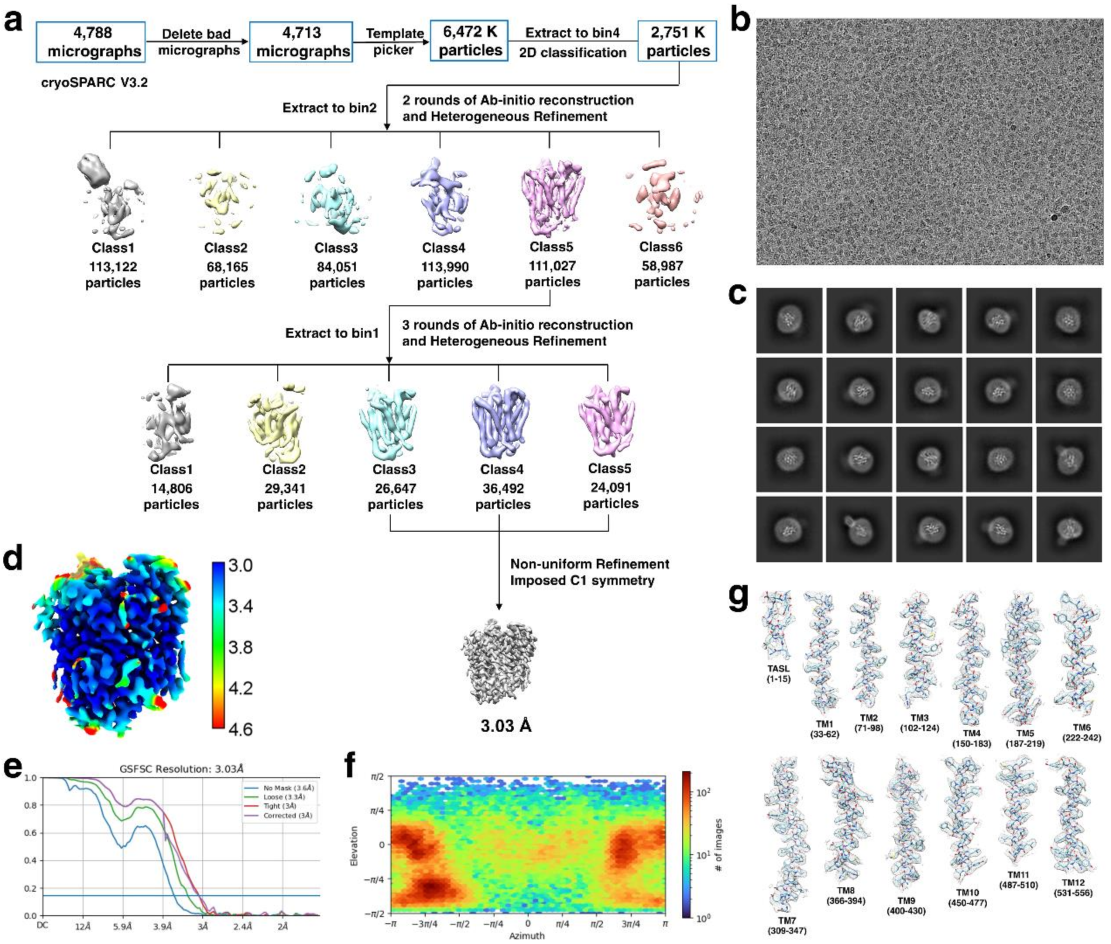

Supplementary Fig. 3 Reconstruction and structure determination of human SLC15A4/TASL(1-20)-EGFP complex

a, The workflow of human SLC15A4/TASL(1-20)-EGFP complex cryo-EM data processing. In brief, 2,751 k particles were kept after 2D classification, and subjected to multiple rounds of ab-initio reconstruction and heterogenous refinement. A final dataset containing 87 k particles were used for Non-uniform refinement and CTF refinement.

b,c, Representative cryo-EM micrograph (**b**) and 2D class averages (**c**) of human SLC15A4/TASL(1-20)-EGFP complex.

d, Local resolution map of the final 3D density map.

e, Gold-standard Fourier Shell correlation (FSC) curve of human SLC15A4/TASL(1-20)-EGFP complex after 3D refinement. The resolution estimation was based on the criterion of FSC 0.143 cutoff.

f, Particle orientation distributions in the last iteration of the structural refinement.

g, Density maps of the transmembrane regions of human SLC15A4 and TASL. Stick style atomic models (blue) were fitted into the cryo-EM density maps (gray mesh). The density maps were contoured at 6σ.

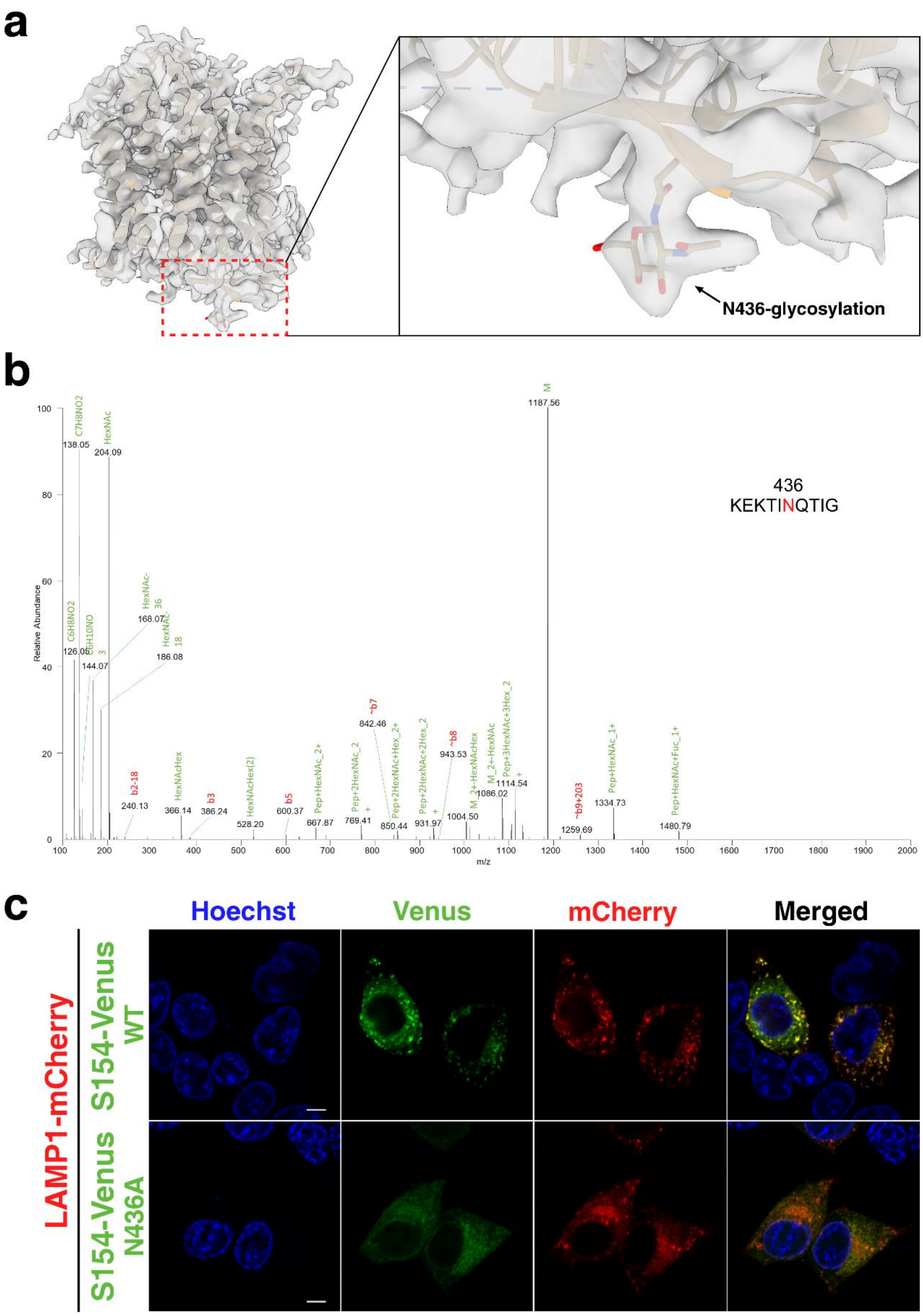

Supplementary Fig. 4 Glycosylation identification of human SLC15A4.

a, Close-up view of N436-glycosylation of human SLC15A4. The density of N436-glycosylation are indicated with red dashed box and shown enlarged.

b, Mass spectrum for glycosylation identification of human SLC15A4 sample.

c, Confocal microscopy images of HEK293T cells co-transfected with lysosome marker LAMP1-mCherry and SLC15A4(WT or N436A)-Venus. Localization of LAMP1 and SLC15A4 are shown. Data are representative of two biological independent experiment. WT, wild type. S154, SLC15A4. Scale bars, 5 μm.

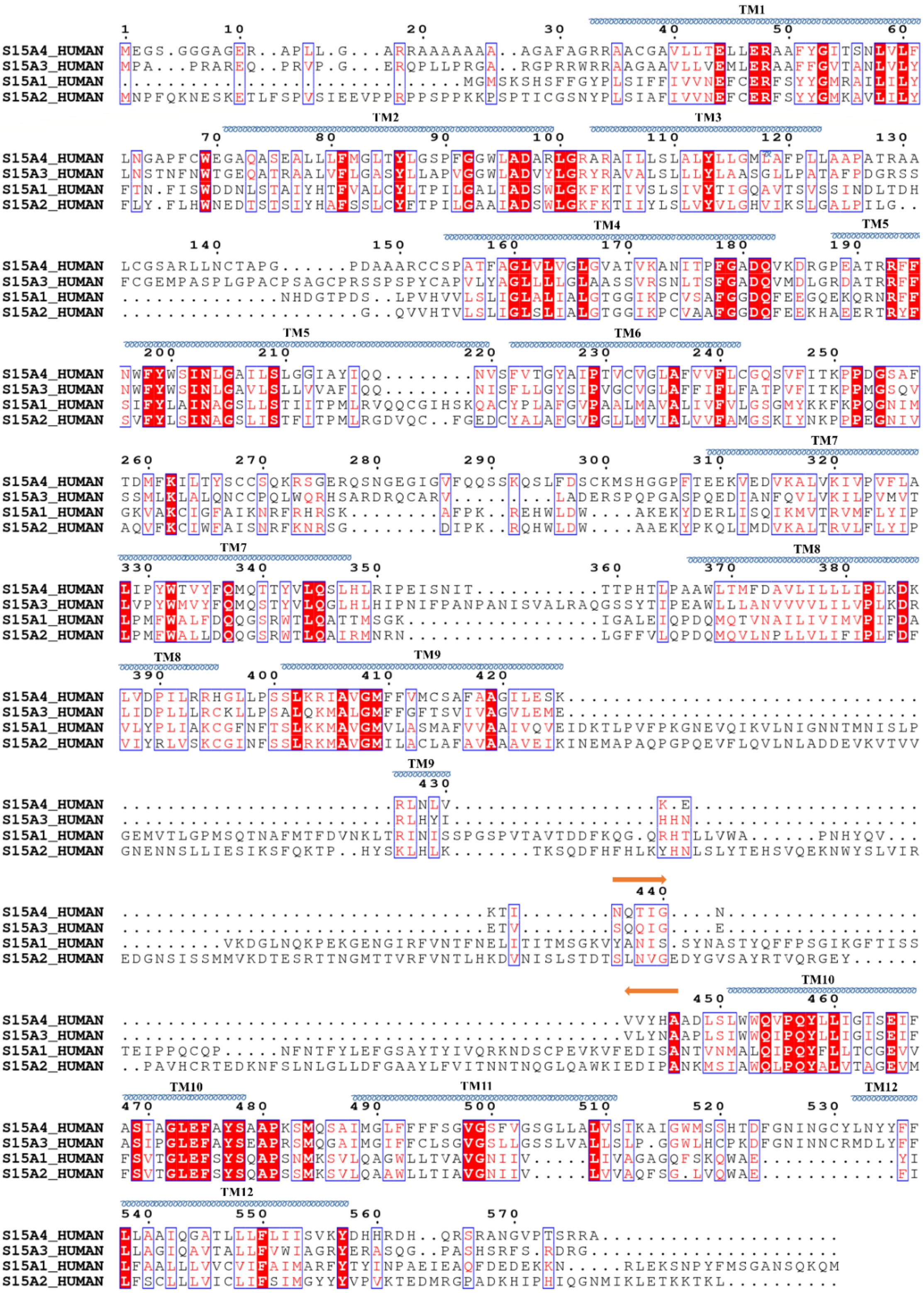

Supplementary Fig. 5 Sequence alignment of human SLC15 family proteins

a, Sequence alignment of human SLC15A1, SLC15A2, SLC15A3 and SLC15A4 using ESPript3. The blue helix and the orange arrows indicated the TM helices and the β strands of the SLC15 family proteins.

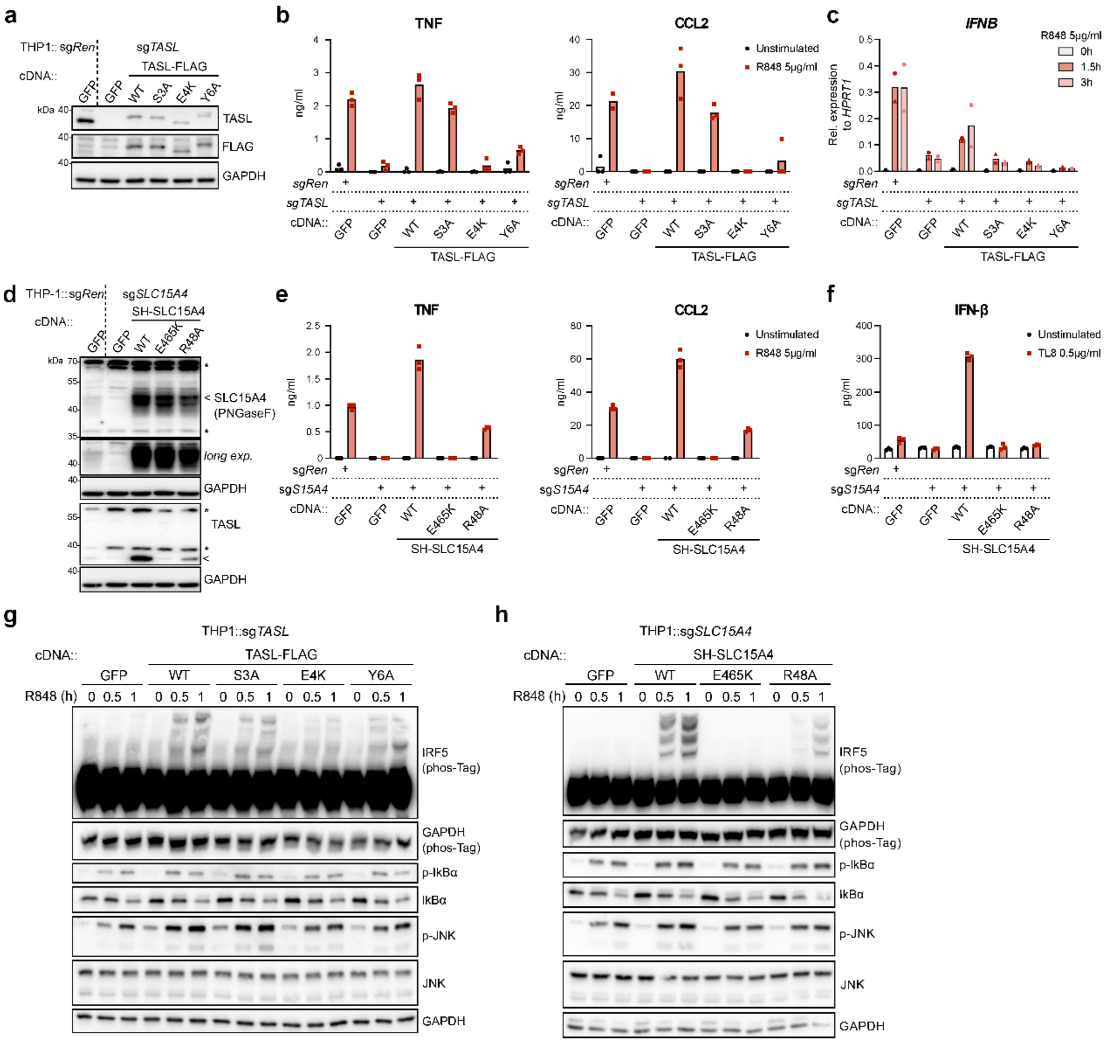

Supplementary Fig. 6 SLC15A4-TASL interaction is essential for endolysosomal TLR7/8-induced IRF5 activation and downstream responses.

a, Immunoblots of control (sg*Ren*) or TASL knockout THP1 cells stably reconstituted with indicated constructs.

b, TNF (left) and CCL2 (right) production by the indicated THP1 cell lines upon R848 stimulation (5 µg/ml, 24 h). Cell supernatants were analyzed by ELISA.

c, *IFNB* mRNA levels of the indicated THP1 cell lines after R848 stimulation (5 µg/ml, for 0-3 h) measured by qPCR.

d, Immunoblots of control (sg*Ren*) or SLC15A4 knockout THP1 cells stably reconstituted with indicated constructs. PNGase F: PNGase F treatment. Arrow: specific signal; Asterisks: unspecific bands.

e, TNF (left) and CCL2 (right) production by the indicated THP1 cell lines upon R848 stimulation (5 μg/ml, 24 h). Cell supernatants were analyzed by ELISA.

f, IFNβ secretion after TL-8 stimulation (0.5 μg/ml, 24 h). Cell supernatants were analyzed by ELISA.

g, h, Immunoblots of THP1 knockout cells reconstituted with the indicated TASL (**g**) and SLC15A4 (**h**) mutant constructs after stimulation with R848 (5 μg/ml for 0-1 h). Data in (**a**, **d**, **g**, **h**) are representative of two-three independent experiments. In **b**, **e**, **f**, data show mean of stimulation replicates from one experiment representative of two independent experiments. Data in (**c**) data show mean of two independent experiments. Source data for relevant information are provided as a Source Data file.

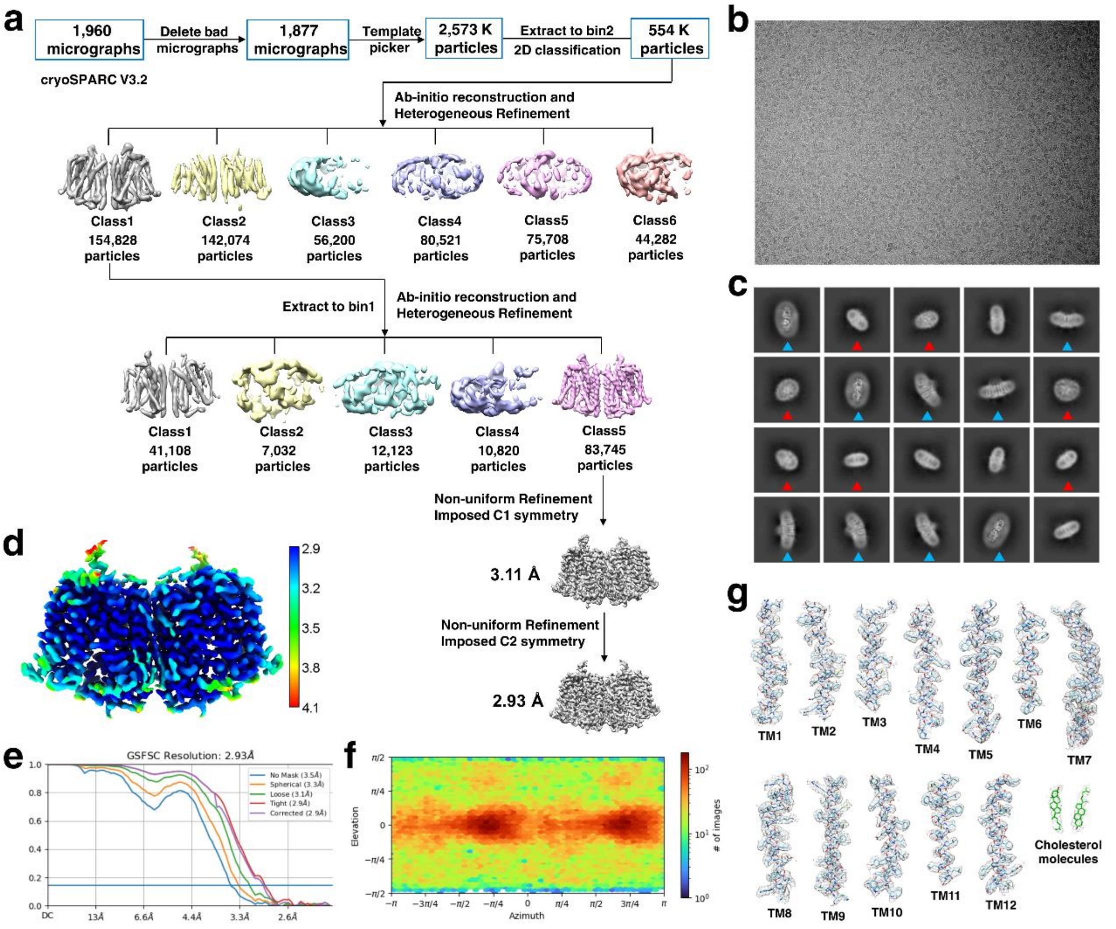

Supplementary Fig. 7 Reconstruction and structure determination of human SLC15A4 in apo state.

a, The workflow of human SLC15A4 cryo-EM data processing. In brief, 554 k particles were kept after 2D classification, and subjected to multiple rounds of ab-initio reconstruction and heterogenous refinement. A final dataset containing 84 k particles were used for Non-uniform refinement and CTF refinement.

b, Representative cryo-EM micrograph of human SLC15A4 in apo state.

c, Representative 2D averages of human SLC15A4 in apo state, the blue and red arrows indicate the dimeric and monomeric particles of SLC15A4, respectively.

d, Local resolution map of the final 3D density map.

e, Gold-standard Fourier Shell correlation (FSC) curve of dimeric SLC15A4 after 3D refinement. The resolution estimation was based on the criterion of FSC 0.143 cutoff.

f, Particle orientation distributions in the last iteration of the structural refinement.

g, Density maps of the transmembrane regions of dimeric SLC15A4 in apo state and the bound cholesterol molecules. Stick style atomic models of SLC15A4 (blue) and cholesterol molecules (green) were fitted into the cryo-EM density maps (gray mesh). The density maps were contoured at 6σ.

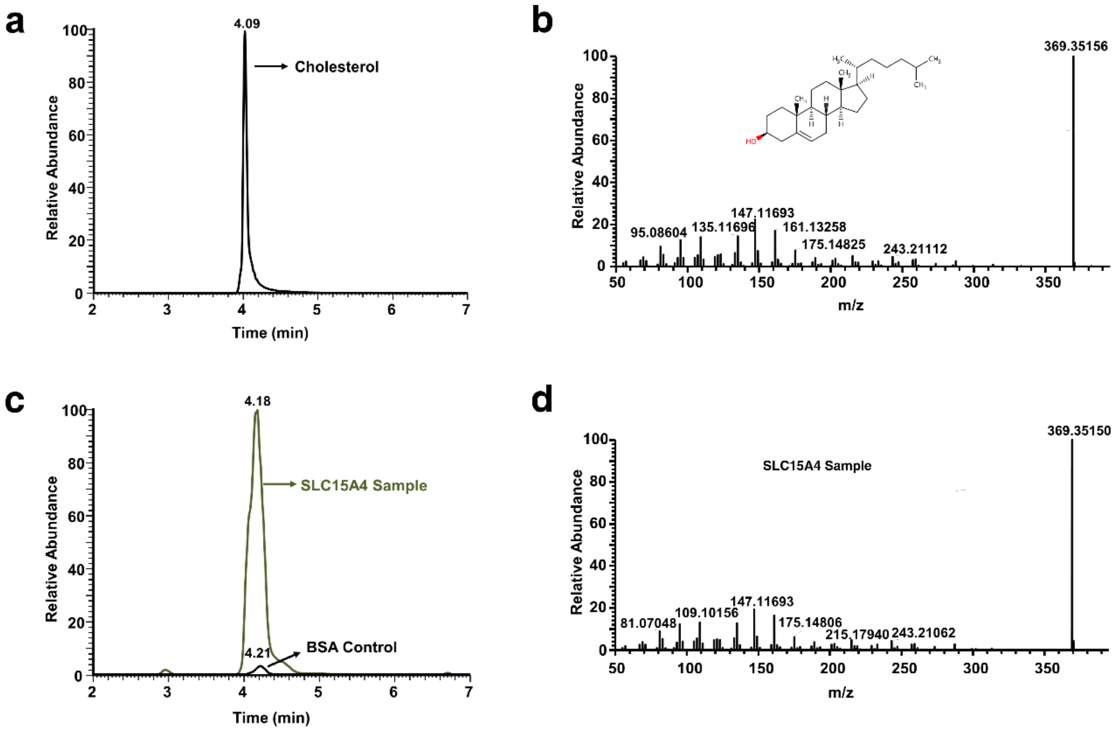

Supplementary Fig. 8 SLC15A4-bound cholesterol identification by mass spectrometry

a-d, The Liquid Chromatography with tandem mass spectrometry (LC-MS-MS) analysis for cholesterol reference standards (**a** and **b**) and SLC15A4 sample (**c** and **d**).

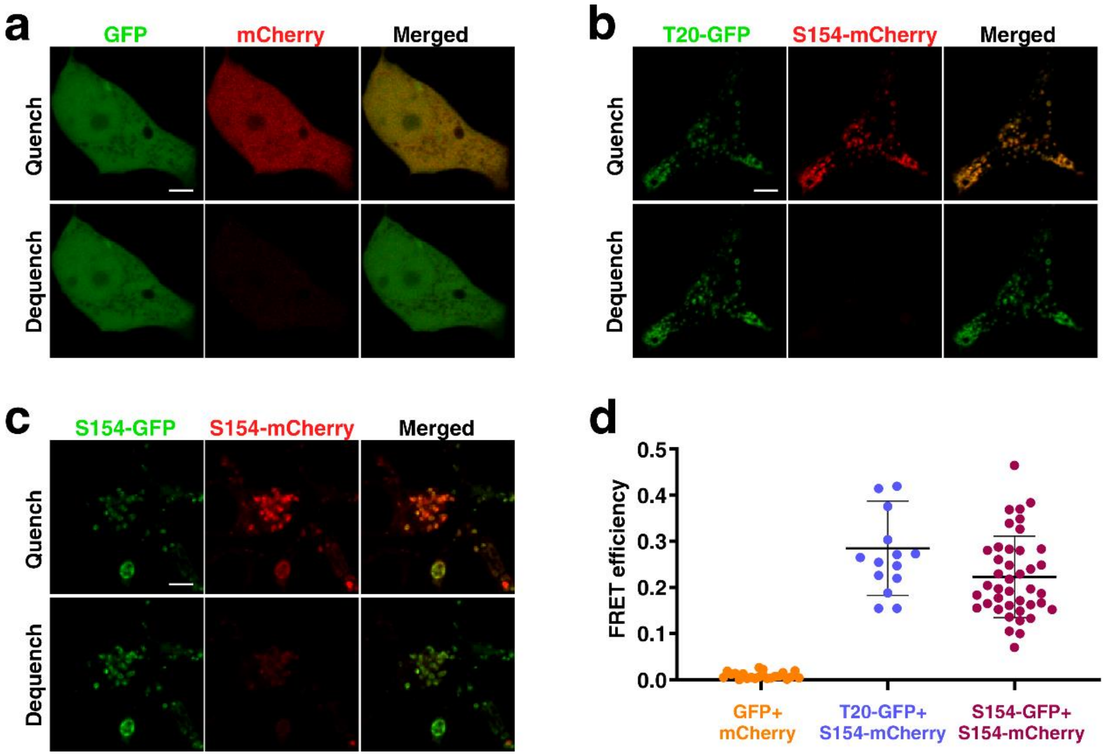

Supplementary Fig. 9 SLC15A4 can form oligomer in vivo.

a-c, Dequencing FRET to measure the FRET efficiency between GFP and mCherry (**a**), between TASL(1-20)-GPF and SLC15A4-mCherry (**b**), between SLC15A4-GFP and SLC15A4-mCherry (**c**) in HEK293T cells. Representative confocal images from 36 (**a**), 14 (**b**) and 41 (**c**) biological independent cells were shown respectively. Scale bars, 5 µm. T20, TASL(1-20). S154, SLC15A4.

d, FRET efficenty in (**a-c**) was calculated and plotted (n = 36 (**a**), 14 (**b**) and 41 (**c**) biological independent experiment, respectively). Bars indicate mean ± s.d. Source data for relevant information are provided as a Source Data file.

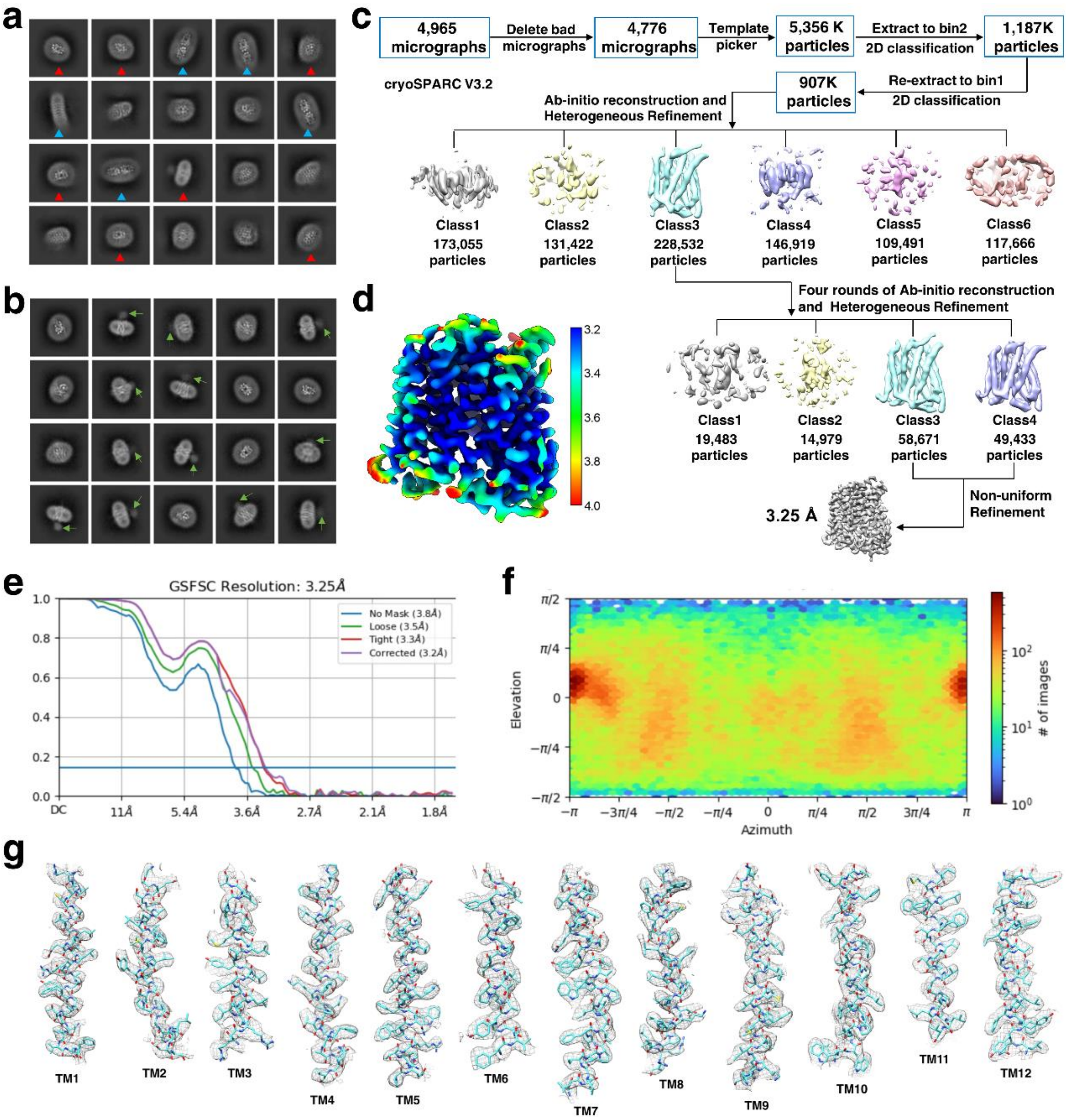

Supplementary Fig. 10 Reconstruction and structure determination of monomeric SLC15A4/ALFA_Nanobody complex

a, The initial 2D averages of SLC15A4/ALFA_Nanobody complex, the blue and red arrows indicate the dimeric and monomeric particles, respectively.

b, The final 2D averages of monomeric SLC15A4/ALFA_Nanobody complex, the green arrow indicate the density of the bound ALFA_Nanobody in each 2D averages.

c, The workflow of human SLC15A4/ALFA_Nanobody complex cryo-EM data processing. In brief, 1,187 k particles were kept after 2D classification, and subjected to multiple rounds of ab-initio reconstruction and heterogenous refinement. A final

dataset containing 108 k particles were used for Non-uniform refinement and CTF refinement.

d, Local resolution map of the final 3D density map.

e, Gold-standard Fourier Shell correlation (FSC) curve of monomeric SLC15A4/ALFA_Nanobody complex after 3D refinement. The resolution estimation was based on the criterion of FSC 0.143 cutoff.

f, Particle orientation distributions in the last iteration of the structural refinement.

g, Density maps of the transmembrane regions of monomeric SLC15A4 in apo state. Stick style atomic models (blue) were fitted into the cryo-EM density maps (gray mesh). The density maps were contoured at 6σ.

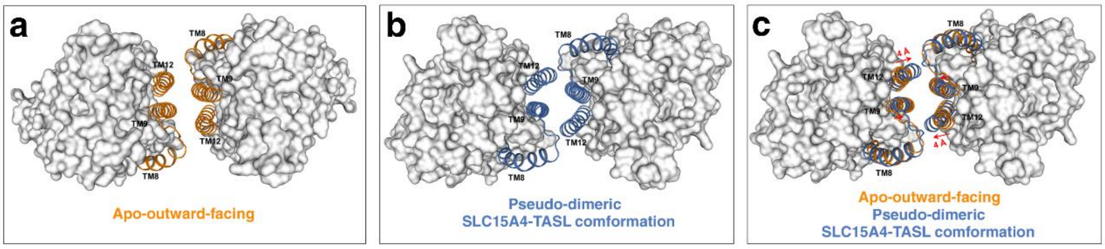

Supplementary Fig. 11 The conformational changes of SLC15A4 during TASL binding breaks the SLC15A4 dimer interface

a,b, Structure of apo SLC15A4 (**a**) and Pseudo-dimeric SLC15A4-TASL (**b**) in surface representation. In particular, the TM helices playing essential role in forming SLC15A4 dimer interface are in ribbon representation and colored orange and blue, respectively.

c, Structure comparison between apo SLC15A4 and Pseudo-dimeric SLC15A4-TASL in (**a**) and (**b**) The red arrows indicate the movement of TM helices.

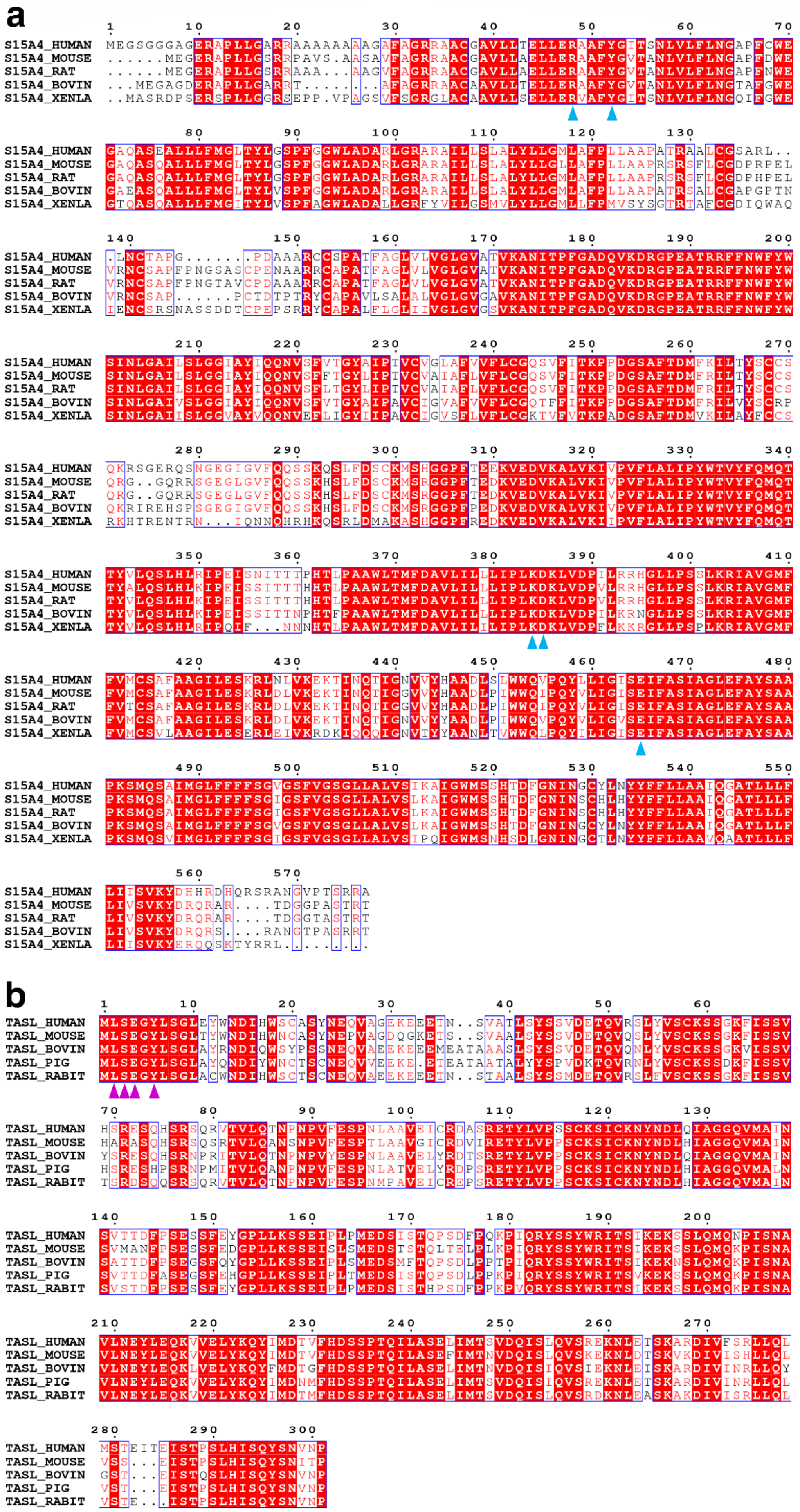

Supplementary Fig. 12 Sequence alignment of SLC15A4 or TASL in different species

a,b, Sequence alignment of SLC15A4 (**a**) or TASL (**b**) in different species using ESPript3. Residues are considered as highly similar are colored in red and framed in blue.The blue and purple arrows indicated the key amino residues of either SLC15A4 or TASL in SLC15A4-TASL interaction, respectively.

Supplementary Tables

Supplementary Table 1. Cryo-EM data collection, refinement and validation statistics

	SLC15A4-TASL complex (EMDB-36753) (PDB 8ZJU)	SLC15A4 dimer (EMDB-36752) (PDB 8JZS)	SLC15A4 Monomer (EMDB-36751) (PDB 8JZR)
**Data collection and processing**			
Magnification	105,000	64,000	105,000
Voltage (kV)	300	300	300
Electron exposure (e–/Å^2)	50	50	50
Defocus range (μm)	-1.0 ~ -2.5	-1.0 ~ -2.5	-1.0 ~ -2.5
Pixel size (Å)	0.8433	1.0979	0.8374
Symmetry imposed	C1	C2	C1
Initial particle images (no.)	6,471,651	2,573,105	5,355,994
Final particle images (no.)	87,230	83,745	108,104
Map resolution (Å)	3.03	2.93	3.25
FSC threshold	0.143	0.143	0.143
Map resolution range (Å)	3.0-4.6	2.9-4.1	3.2-4.0
**Refinement**			
Initial model used (PDB code)	*de novo,* AlphaFold	*de novo,* AlphaFold	PDB 8JZS
Model resolution (Å)	3.1	2.9	3.2
FSC threshold	0.143	0.143	0.143
Model resolution range (Å)	2.98-3.20	2.87-3.06	3.21-3.50
Map sharpening *B* factor (Å^2)	109.0	111.6	139.4
Model composition			
Non-hydrogen atoms	3727	7500	3474
Protein residues	488	972	456
Ligands	0	4	0
B factors (Å^2)			
Protein	55.84	35.16	31.31
Ligand	0	22.0	0
R.m.s. deviations			
Bond lengths (Å)	0.005	0.007	0.007
Bond angles (°)	0.988	1.129	1.071
Validation			
MolProbity score	1.39	1.85	1.59
Clashscore	6.61	10.14	5.53
Poor rotamers (%)	0	0	0
Ramachandran plot			
Favored (%)	97.9	95.38	95.74
Allowed (%)	2.1	4.2	3.81
Disallowed (%)	0	0.42	0.45

Supplementary Table 2. Plasmids and primer sequences information

Plasmids	Primer secquences
pCDNA3.1(+)-SLC15A4-Strep	F:GCTGGCTAGCGTTTAAACTTAAGCTTGCCACCATGGAGGGATCCGGAGGAGGAGCT<br>R:GACCCTGGAAGAGAACCTCCAGGGATCCTGCTCTCCGAGAGGTAGGGACACCAT
pCDNA3.1(+)-SLC15A4-ALFA-Strep	F:CTGGAGGAGGAGCTGAGAAGAAGACTGACAGAGCCATTCACCGAGGAAAAAGTTG<br>R:CTTCTTCTCAGCTCCTCCTCCAGTCTGCTGGGCTTGGTAATGAACACGCT
pCAG-TASL(1-20)-GFP-Strep	F:GTGCTGTCTCATCATTTTGGCAAAGAATTCGCCACCATGCTGTCAGAAGGGTATCT<br>R:GAACAGCTCCTCGCCCTTGCTCACACAACTCCAGTGGATGTCATTC
pCDNA3.1(+)-SLC15A4	F:GCTGGCTAGCGTTTAAACTTAAGCTTGCCACCATGGAGGGATCCGGAGGAGGAGCT<br>R:CTGTGCTGGATATCTGCAGAATTCTCATGCTCTCCGAGAGGTAGGGACACCAT
pCDNA3.1(+)-SLC15A4-mCherry	F:GTCCCTACCTCTCGGAGAGCAGGAGGTACCGGTTACCCATACGATGTT<br>R:CCCTGGAAGAGAACCTCCAGGGATCCCTTGTACAGCTCGTCCATG
pCAG-CMV-TASL-GFP	F: GCGGAGGCAGCAAAATCGAAGGAATGGTGAGCAAGGGCGAG<br>R:GAAGAGAACCTCCAGGGATCCCTTGTACAGCTCGTCCATGC
pCAG-UBC-TASL-GFP	F:GGCCGTTTTTGGCTTTTTTGTTAGACGCGGCCGCGCCACCATGCTGAGCGAGGGCTAT<br>R:ATTTCTCAGTATAGCAATGTAAATCCAGGCGGAGGCAGCGACTATAAGGACGACG
pCAG-TASL(1-20)-GFP	F:GCTGTCTCATCATTTTGGCAAAGAATTCGCCACCATGCTGTCAGAAGGGTAT<br>R:CCTGGAAGAGAACCTCCAGGGATCCCTTGTACAGCTCGTCCAT
pCAG-TASL(1-20&207-301)-GFP	F: GGAATGACATCCACTGGAGTTGTAATGCAGTTCTGAATGAGTAC<br>R:CCTTGCTCACCATTCCTTCGATTTTGCTGCCTCCGCCTGGATTTACATTGCT
pCAG-TASL(1-215)-GFP	F:GCTGTCTCATCATTTTGGCAAAGAATTCGCCACCATGCTGTCAGAAGGGTAT<br>R:CCTTCGATTTTGCTGCCTCCGCCCTCCAGGTACTCATTCAGAACT
pLVX-EFS-SLC15A4-Hygro	F:GGACCGGTACTAGTGCCACCATGGAGGGATCCGGAGGAGGAGCT<br>R:GAGAGGGGCGGGATCCGCGGCCGCTCATGCTCTCCGAGAGGTAG
pLVX-UBC-TASL-Hygro	F:GGCTTTTTTGTTAGACGCGGCCGCGCCACCATGCTGAGCGAGGGCTATCT<br>R:CGTCGTCCTTATAGTCGCTGCCTCCGCCTGGATTTACATTGCTATACTGAGAAAT

Supplementary References

1 Baek, M. *et al.* Accurate prediction of protein structures and interactions using a three-track neural network. *Science* **373**, 871-876, doi:10.1126/science.abj8754 (2021).

2 Li, X., Romero, P., Rani, M., Dunker, A. K. & Obradovic, Z. Predicting Protein Disorder for N-, C-, and Internal Regions. *Genome Inform Ser Workshop Genome Inform* **10**, 30-40 (1999).
