# Supplementary material for: Structural basis for recruitment of TASL by SLC15A4 in human endolysosomal TLR signaling
Source: Nat Commun. 2023 Oct 20;14:6627. doi: 10.1038/s41467-023-42210-9 (PMC10589346; doi:10.1038/s41467-023-42210-9)
Supplement: Supplementary file 3 — Reporting Summary [file 41467_2023_42210_MOESM3_ESM.pdf]

nature portfolio

Corresponding author(s): Maojun Yang

Last updated by author(s): Sep 21, 2023

Reporting Summary

Nature Portfolio wishes to improve the reproducibility of the work that we publish. This form provides structure for consistency and transparency in reporting. For further information on Nature Portfolio policies, see our Editorial Policies and the Editorial Policy Checklist.

Statistics

For all statistical analyses, confirm that the following items are present in the figure legend, table legend, main text, or Methods section.

n/a	Confirmed	
☐	☒	The exact sample size (*n*) for each experimental group/condition, given as a discrete number and unit of measurement
☐	☒	A statement on whether measurements were taken from distinct samples or whether the same sample was measured repeatedly
☒	☐	The statistical test(s) used AND whether they are one- or two-sided<br>*Only common tests should be described solely by name; describe more complex techniques in the Methods section.*
☒	☐	A description of all covariates tested
☒	☐	A description of any assumptions or corrections, such as tests of normality and adjustment for multiple comparisons
☐	☒	A full description of the statistical parameters including central tendency (e.g. means) or other basic estimates (e.g. regression coefficient) AND variation (e.g. standard deviation) or associated estimates of uncertainty (e.g. confidence intervals)
☒	☐	For null hypothesis testing, the test statistic (e.g. *F*, *t*, *r*) with confidence intervals, effect sizes, degrees of freedom and *P* value noted<br>*Give P values as exact values whenever suitable.*
☒	☐	For Bayesian analysis, information on the choice of priors and Markov chain Monte Carlo settings
☒	☐	For hierarchical and complex designs, identification of the appropriate level for tests and full reporting of outcomes
☒	☐	Estimates of effect sizes (e.g. Cohen's *d*, Pearson's *r*), indicating how they were calculated

Our web collection on statistics for biologists contains articles on many of the points above.

Software and code

Policy information about availability of computer code

Data collection	Cryo-EM data collection: AutoEMation2.0<br>Confocal Images collection: NIS-Elements (the built-in software of Nikon A1HD25 microscope)<br>Western blot data detection: ImageQuant TL 7.0; Vilber Fusion Solo S7, Viber EvolutionCapt-v18.12<br>Elisa data collection: PerkinElmer EnVision Multilabel Reader ; SoftMax Pro v 7.0; BioTekGen5 Software v2.0.9
Data analysis	For cryo-EM data processing: cryoSPARC V3.2, PHENIX1.14-3374, ResMap1.1.4;<br>For cryo-EM atomic model refinement and analysis: Coot0.8.9, PHENIX1.14-3374, MolProbity (part of PHENIX package);<br>For cryo-EM figure preparation: PyMol2.1.0, Chimera1.14, ChimeraX1.25;<br>For protein sequence analysis: ESPpript3<br>For assay data representation and statistical analysis: GraphPad Prism Version 8.2.1<br>For Confocal images analysis: NIS-Elements Viewer 4.50<br>Mass spectrometry data analysis: Xcalibur version 4.3.73.11 and version 4.4

For manuscripts utilizing custom algorithms or software that are central to the research but not yet described in published literature, software must be made available to editors and reviewers. We strongly encourage code deposition in a community repository (e.g. GitHub). See the Nature Portfolio guidelines for submitting code & software for further information.

Data

Policy information about availability of data

All manuscripts must include a data availability statement. This statement should provide the following information, where applicable:

- Accession codes, unique identifiers, or web links for publicly available datasets
- A description of any restrictions on data availability
- For clinical datasets or third party data, please ensure that the statement adheres to our policy

The 3D cryo-electron microscopy density map and the coordinates of atomic models has been deposited in the Electron Microscopy Data Bank (EMDB) and the Protein Data Bank (PDB) with the following accession codes: EMD-36753 [https://www.ebi.ac.uk/pdbe/entry/emdb/EMD-36753] and 8JZU [http://doi.org/10.2210/pdb8jzu/pdb] for SLC15A4-TASL complex ; EMD-36752 [https://www.ebi.ac.uk/pdbe/entry/emdb/EMD-36752] and 8JZS [http://doi.org/10.2210/pdb8jzs/pdb] for SLC15A4 apo dimer state; EMD-36751 [https://www.ebi.ac.uk/pdbe/entry/emdb/EMD-36751] and 8JZR [http://doi.org/10.2210/pdb8jzr/pdb]for SLC15A4 apo monomer state. The other structures used for comparative analysis in this study can be found in the PDB with the following accession codes: 7PMW [http://doi.org/10.2210/pdb7pmw/pdb], 7PMX [http://doi.org/10.2210/pdb7pmx/pdb] and 7PN1 [http://doi.org/10.2210/pdb7pn1/pdb] for SLC15A1; 7NQK [http://doi.org/10.2210/pdb7nqk/pdb] and 7PMY [http://doi.org/10.2210/pdb7pmy/pdb] for SLC15A2. All protein sequences used in this study are available at Uniprot (https://www.uniprot.org/) with the following accession codes: Q8N697 for human SLC15A4; Q9HAI6 for human TASL; P46059 for Human SLC15A1; Q16348 for Human SLC15A2; Q8IY34 for Human SLC15A3; Q91W98 for mouse SLC15A4; O09014 for rat SLC15A4; A6QQL0 for bovin SLC15A4; Q68F72 for African clawed fog SLC15A4; Q9D3J9 for mouse TASL; Q32LD7 for bovin TASL; F1SPV1 for pig TASL; G1U138 for rabbit TASL. All other data are available in the manuscript or in the supplementary materials. Source data of relevant information are provided as source data files.

Field-specific reporting

Please select the one below that is the best fit for your research. If you are not sure, read the appropriate sections before making your selection.

☒ Life sciences ☐ Behavioural & social sciences ☐ Ecological, evolutionary & environmental sciences

For a reference copy of the document with all sections, see nature.com/documents/nr-reporting-summary-flat.pdf

Life sciences study design

All studies must disclose on these points even when the disclosure is negative.

Sample size	No statistical method was used to predetermine sample size.
Data exclusions	In cryo-EM data processing in cryoSPARC, the low-quality data such as bad micrographs or particles were excluded to reach high-resolution using statistical methods. The exclusion algorithm is as implemented in cryoSPARC.
Replication	Reported experiments were repeated at least 2 times with comparable results.
Randomization	Randomization is not relevant to the majority of experiments of this study, because protein samples are not required to be allocated into experimental groups in the biochemical studies. Randomization was used only in cryo-EM image processing and structure refinement.
Blinding	The researchers were not blinded to group allocation, because samples must be grouped in mutagenesis analysis (WT vs mutations for expression and transfection) and immunoblotting (different antibodies used) during the experimental performances.

Reporting for specific materials, systems and methods

We require information from authors about some types of materials, experimental systems and methods used in many studies. Here, indicate whether each material, system or method listed is relevant to your study. If you are not sure if a list item applies to your research, read the appropriate section before selecting a response.

Materials & experimental systems

n/a	Involved in the study	
☐	☒	Antibodies
☐	☒	Eukaryotic cell lines
☒	☐	Palaeontology and archaeology
☒	☐	Animals and other organisms
☒	☐	Human research participants
☒	☐	Clinical data
☒	☐	Dual use research of concern

Methods

n/a	Involved in the study	
☒	☐	ChIP-seq
☒	☐	Flow cytometry
☒	☐	MRI-based neuroimaging

Antibodies

Antibodies used	Primary antibodies used in this study with supplier and catalog number<br>StrepTag Mouse Monoclonal Antibody, Easybio, Cat# BE2076, Dilution: 1:3000;<br>FlagTag Mouse Monoclonal Antibody, Easybio, Cat# BE2004, Dilution: 1:3000;<br>Human CXorf21 (TASL) Mouse polyclonal Antibody, Abcam, Cat# ab69152, Dilution: 1:1000;<br>Human SLC15A4 Rabbit Polyclonal Antibody, Abmart Cat# PHC6279, Dilution: 1:500;<br>custom made rabbit anti-SLC15A4 Antibody, (Genscript, described in Heinz et al 2020, ref. #5), Dilution: 1:1000;<br>custom made rabbit anti-TASL Antibody (Eurogentec, described in Zhang et al. 2023 Cell reports, ref. #63), Dilution: 1:1000;<br>rabbit anti-SAPK/JNK Antibody, Cell signaling, Cat# 9252, Dilution: 1:1000;<br>rabbit anti-phospho-SAPK/JNK Antibody, Cell signaling, Cat# 4668, Dilution: 1:1000;<br>mouse anti-IkBα Antibody, Cell signaling, Cat# 4814, 1:1000;<br>rabbit anti-phospho-IkBα Antibody, Cell signaling, Cat# 2859, 1:1000;<br>mouse anti-Flag M2 Antibody, Sigma, Cat# F1804, 1:1000;<br>rabbit anti-TASL(CXorf21) Antibody, Sigma, Cat# HPA001185, 1:1000;<br>rabbit anti-IRF5 Antibody, Abcam, Cat# ab181553, 1:1000;<br>mouse anti-GAPDH Antibody, Santa Cruz, Cat# sc-365062, 1:1000.
Validation	Specificity of custom made rabbit anti-SLC15A4 (Genscript) and anti-TASL (TASL HM, Eurogentec) have been validated previously (Heinz et al., Ref. #5 and Zhang et al., Ref. #63). All other antibodies were purchased from commercial vendors and validation for indicated species. We refer to the supplier's websites and datasheets to find statements on specificity and dilution for the use of the antibodies:<br>StrepTag Mouse Monoclonal Antibody; http://www.bioeasytech.com/product/2442.html?goods_id=4427<br>FlagTag Mouse Monoclonal Antibody; http://www.bioeasytech.com/product/2383.html?goods_id=4368<br>Human CXorf21 Mouse polyclonal Antibody; https://www.abcam.com/cxorf21-antibody-ab69152.html<br>Human SLC15A4 Rabbit Polyclonal Antibody; http://www.ab-mart.com.cn/page.aspx?node=%2077%20&id=%2062120<br>rabbit anti-SAPK/JNK Antibody; https://www.cellsignal.com/products/primary-antibodies/sapk-jnk-antibody/9252<br>rabbit anti-phospho-SAPK/JNK Antibody; https://www.cellsignal.com/products/primary-antibodies/phospho-sapk-jnk-thr183-tyr185-81e11-rabbit-mab/4668<br>mouse anti-IkBα Antibody; https://www.cellsignal.com/products/primary-antibodies/ikba-l35a5-mouse-mab-amino-terminal-antigen/4814<br>rabbit anti-phospho-IkBα Antibody; https://www.cellsignal.com/products/primary-antibodies/phospho-ikba-ser32-14d4-rabbit-mab/2859<br>mouse anti-Flag M2 Antibody; https://www.sigmaaldrich.com/product/sigma/f1804<br>rabbit anti-TASL Antibody; https://www.sigmaaldrich.com/product/sigma/hpa001185<br>rabbit anti-IRF5 Antibody; https://www.abcam.com/products/primary-antibodies/irf5-antibody-epr17067-ab181553.html<br>mouse anti-GAPDH Antibody; https://www.scbt.com/zh/p/gapdh-antibody-g-9/

Eukaryotic cell lines

Policy information about cell lines

Cell line source(s)	HEK293T cell line was obtained from ATCC, Cat# CRL-3216; FreeStyle™ 293-F Cell line were purchased from Invitrogen, Cat# R790-07; THP1 cell line was obtained from ATCC, Cat# TIB-202.
Authentication	HEK293T cells and THP1 cells were authenticated by STR profiling. FreeStyle™ 293-F Cells for protein expression were not authenticated.
Mycoplasma contamination	The cell line tested was negative for mycoplasma contamination.
Commonly misidentified lines<br>(See ICLAC register)	No commonly misidentified cell lines were used.
